# Plasma LncRNA-ATB, a Potential Biomarker for Diagnosis of Patients with Coal Workers’ Pneumoconiosis: A Case-Control Study

**DOI:** 10.3390/ijms17081367

**Published:** 2016-08-22

**Authors:** Jixuan Ma, Xiuqing Cui, Yi Rong, Yun Zhou, Yanjun Guo, Min Zhou, Lili Xiao, Weihong Chen

**Affiliations:** 1 Department of Occupational & Environmental Health, School of Public Health, Tongji Medical College, Huazhong University of Science and Technology, Wuhan 430030, China; xjmajixuan@163.com (J.M.); cuixiuqing2008@163.com (X.C.); sarayunzhou@gmail.com (Y.Z.); hustghkan@126.com (Y.G.); mzhou90@126.com (M.Z.); Yeyushunong@163.com (L.X.); 2 Key Laboratory of Environment and Health, Ministry of Education & Ministry of Environmental Protection, and State Key Laboratory of Environmental Health (Incubating), School of Public Health, Tongji Medical College, Huazhong University of Science and Technology, Wuhan 430030, China; 3 Long Hua Center for Disease Control and Prevention, Shenzhen 518109, China; rongyi1007@126.com

**Keywords:** coal workers’ pneumoconiosis (CWP), long noncoding RNA, lncRNA-ATB, epithelial-mesenchymal transition (EMT), biomarker

## Abstract

LncRNA-ATB (lncRNA was activated by transforming growth factor-β) has been reported to be involved in specific physiological and pathological processes in human diseases, and could serve as biomarkers for cancers. However, the role of lncRNA-ATB in coal workers’ pneumoconiosis (CWP) is still unknown. This study aimed to investigate the association between lncRNA-ATB and CWP. Quantitative real-time polymerase chain reaction was performed to detect plasma lncRNA-ATB expression in 137 CWP patients, 72 healthy coal miners and 168 healthy controls. LncRNA-ATB was significantly upregulated in CWP (*p* < 0.05). Compared with the healthy controls and healthy coal miners, the odds ratios (ORs) (95% confidence interval (CI)) for CWP were 2.57 (1.52–4.33) and 2.17 (1.04–4.53), respectively. LncRNA-ATB was positively associated with transforming growth factor-β1 (TGF-β1) (*r* = 0.30, *p* = 0.003) and negative correlated with vital capacity (VC) (*r* = −0.18, *p* = 0.033) and forced vital capacity (FVC) (*r* = −0.18, *p* = 0.046) in CWP patients. Compared with healthy controls, the area under the curve (AUC) was 0.84, resulting in a 71.17% sensitivity and 88.14% specificity. When compared with healthy coal miners, the AUC was 0.83, the sensitivity and specificity were 70.07% and 86.36%, respectively. LncRNA-ATB expression is commonly increased in CWP and significantly correlates with the TGF-β1 in CWP patients. Furthermore, elevated lncRNA-ATB was associated with CWP risk and may serve as a potential biomarker for CWP.

## 1. Introduction

Coal workers’ pneumoconiosis (CWP), identified by pulmonary parenchyma fibrosis, is a chronic occupational lung disease caused by long-term inhalation of dust in the workplace [1]. Pathologic features of CWP involve focal collections of dust and reticulin around the small airways, fibrotic lesions exhibiting irregularly arranged collagen, and lesions of massive fibrosis [2,3]. Although prevention efforts have been required to implement for decades, CWP is still one of high incidence occupational diseases worldwide, especially in China [4]. Currently, periodic medical screenings for pneumoconiosis normally include chest radiography and spirometry. However, abnormal signs of both tests are often displayed in the late course of the underlying disease. Many workers could not get timely diagnoses and lost opportunities for prevention or treatment. Therefore, novel potential biomarkers for detecting CWP deserved more attention.

Long non-coding RNAs are types of transcripts that are greater than 200 nucleotides in length, can exert their biological functions by binding to RNA, DNA and protein, and often do not have the capability to coding proteins [5,6]. LncRNAs have recently been found to be involved in specific physiological and pathological processes in a wide range of human diseases, and can be stable in the plasma and other body fluids [7,8,9,10]. Therefore, lncRNAs could serve as biomarkers for the diagnosis and poor prognosis of human diseases, such as cancers and lung fibrosis [11,12,13]. For example, a high expression of lncRNA-ATB participated in the development of colorectal cancer (CRC) and was a novel indicator of poor prognosis in patients with CRC [14]. Low expression of lncRNA-ATB plays a critical role in pancreatic cancer progression and prognosis, and may serve as a potential prognostic biomarker in pancreatic cancer patients [15]. However, the study investigated the role of lncRNAs in CWP is limited.

LncRNA-ATB, which was named lncRNA, was activated by transforming growth factor-β (TGF-β). LncRNA-ATB can induce epithelial-mesenchymal transition (EMT) and promote invasion via competitively binding and sequestering the miR-200 family in hepatocellular carcinoma (HCC) [16]. It is widely recognized that EMT regulated by TGF-β is considered a critical signaling pathway in organ fibrosis process [17,18,19,20]. In our previous study, we found that silica-induced fibrosis was regulated by EMT, which was activated by the upregulated TGF-β1 [21]. Moreover, a genome-wide analysis also showed that the miR-200 family was an aberrant expression in CWP [22]. However, the relationship between lncRNA-ATB and CWP remains unclear.

In this study, we hypothesize that lncRNA-ATB may play a potential role in CWP. A case-control study was designed to determine the expression of lncRNA-ATB in human plasma for groups with or without CWP. The objectives of this study were to investigate the association between lncRNA-ATB expression and CWP, and to assess the specificity and sensitivity of using plasma lncRNA-ATB as a potential diagnostic tool for detection of CWP.

## 2. Results

### 2.1. LncRNA-ATB Expression in the Plasma of All Participants

**Figure 1:**
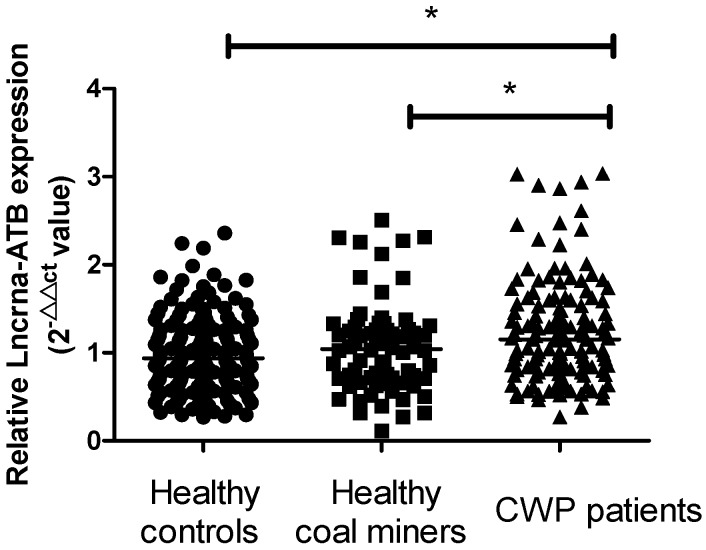
Scatter plots of lncRNA-ATB expressions in different groups. Solid circles, healthy controls; Solid squares, healthy coal miners; Solid triangles, CWP patients. (* *p* < 0.05).

The basic characteristics for all participants were shown in Table 1. This case-control study included 137 patients with CWP patients, 72 were healthy coal miners, and 168 were healthy controls. Subjects with CWP had significantly higher plasma TGF-β1, matrix metalloproteinase-9 (MMP-9), matrix metalloproteinase-2 (MMP-2), Collagen I (Col-1) and Collagen III (Col-3) (*p* < 0.05) when compared with both healthy groups. As expected, in comparison with healthy controls and healthy coal miners, CWP patients had remarkably lower levels of spirometry parameters (percent of predicted forced vital capacity (% PRED FVC), percent of predicted forced expiratory volume in 1 s (% PRED FEV1), and % FEV1/FVC) (*p* < 0.05). The distribution of net years in dust was difference between healthy coal miners and CWP patients. Compared with healthy controls and healthy coal miners, lncRNA-ATB was a higher expression in the plasma of CWP patients (*p* < 0.05) (Figure 1).

### 2.2. Association between LncRNA-ATB Expression and Coal Workers’ Pneumoconiosis (CWP)

The association of lncRNA-ATB and CWP was shown in Table 2. Compared with healthy controls, single factor logistic regression analysis showed a positive relationship between lncRNA-ATB and the CWP. The association was still strongly after adjusting multiple potential confounders. Compared with the subjects in the lowest group of lncRNA-ATB, the multi-variate adjusted odds ratios (ORs) (95% confidence interval (CI)) for CWP were 1.41 (0.73–2.72) and 2.39 (1.29–4.42) from the second group to the third group of lncRNA-ATB. In the secondary analysis, compared with healthy coal miners, lncRNA-ATB was strongly associated with CWP risk with adjusting potential confounders. The OR (95% CI) for CWP was 2.17 (1.04–4.53) for a one-unit increase in log lncRNA-ATB.

### 2.3. Relationship between LncRNA-ATB Expression and Clinical/Biological Features in CWP Patients

Our results showed that plasma TGF-β1, MMP-9 and Col-1 were closely related to spirometry parameters in patients (*p* < 0.05). LncRNA-ATB was positively associated with TGF-β1 (Spearman correlation coefficient *r* = 0.30, *p =* 0.003) and negatively correlated with VC (% PRED VC) (*r = −*0.18, *p =* 0.033) and FVC (% PRED FVC) (*r = −*0.18, *p =* 0.046) (Table 3).

### 2.4. Plasma LncRNA-ATB Expression Can Be a Potential CWP Biomarker

**Figure 2:**
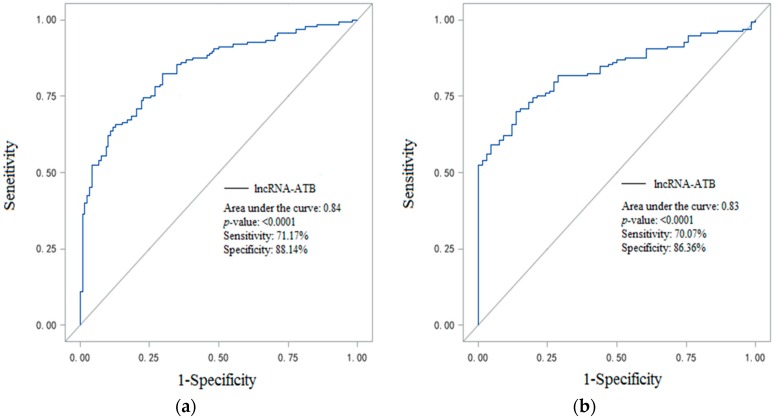
Receiver operating characteristic (ROC) curve analysis for lncRNA-ATB in plasma adjusted for risk factors; (**a**) Compared with healthy controls; and (**b**) compared with healthy coal miners.

Receiver Operating Characteristic (ROC) curve analysis was used to evaluate the discriminatory power of lncRNA-ATB in plasma. We adjusted for age, body mass index (BMI), systolic and diastolic blood pressure (BP). Compared with healthy controls, we found that the area under the curve (AUC) was 0.84 with a cutoff value greater than 0.92, resulting in 71.17% sensitivity and 88.14% specificity. When compared with healthy coal miners, the AUC was 0.83 after adjusting for age and BMI, with a cutoff of greater than 0.70. The sensitivity and specificity were 70.07% and 86.36%, respectively (Figure 2).

## 3. Discussion

In this study, our data clearly demonstrated that an increase in lncRNA-ATB expression level in CWP compared with healthy controls and healthy coal miners. Higher lncRNA-ATB levels were associated with elevated odds of CWP after adjusted for a wide range of risk factors. In CWP patients, lncRNA-ATB had a significantly positive correlation with TGF-β1 and negatively associated with VC (% PRED VC) and FVC (% PRED FVC). Moreover, ROC curve analysis showed that lncRNA-ATB distinguishes patients with CWP from healthy controls and healthy coal miners, and the AUCs were 0.84 and 0.83, respectively.

Long non-coding RNAs are types of transcripts that are greater than 200 nucleotides in length [5,23]. Several previous studies have shown that lncRNAs could serve as key regulators of important biological processes, such as proliferation apoptosis, or cell migration [24,25]. LncRNA-ATB is a novel lncRNA that was first profiled by Yuan et al. in HCC cells [16]. LncRNA-ATB was activated by TGF-β, increased ZEB1 and ZEB2 mRNA and protein levels through competitively binding and sequestering miR-200s family and then induce EMT [16]. Altered expression of lncRNA-ATB has been documented in some human diseases, such as HCC, CRC and pancreatic cancer.

In this study, we found that increased lncRNA-ATB were associated with elevated odds of having CWP. Compared with healthy controls and healthy coal miners, higher lncRNA-ATB expression had higher ORs of CWP after adjusted for the potential confounders. Similarly, Saito et al. found that gastric cancer (GC) patients in the high lncRNA-ATB group had a significantly worse prognosis than patients in the low lncRNA-ATB group [26]. The elevated expression of lncRNA-ATB was associated with tumor stages, histological grade and distant metastasis in renal cell carcinoma [27]. In colon cancer patients, increased lncRNA-ATB played an important role in disease recurrence and decreased survival [28].

To our knowledge, this study is the first report about the expression of lncRNA-ATB in CWP patients. Currently published papers indicated that lncRNA-ATB was activated by TGF-β, involved in the EMT signaling pathway, which may be participated in the progression and prognosis of CWP. It is widely recognized that EMT regulated by TGF-β is considered an important signaling pathway in lung fibrosis process [29,30]. The previous study in our group found that silica-induced fibrosis was regulated by EMT, and the upregulated TGF-β1 was involved in the process of EMT [21]. In this study, TGF-β1 was significantly higher in CWP patients and positively correlated with lncRNA-ATB. These results suggested that upregulated lncRNA-ATB probably influences the process of CWP through a TGF-β-mediated EMT signaling pathway. Similarly, Zhu et al. found lncRNA-ATB, a transcriptional activator of TGF-β, was overexpressed and associated with miR-200c in keloid fibroblasts [31]. In our study, we matched the average net years in dust between healthy coal miners and CWP patients. However, there was still a difference in distributions between two groups. The major reason was that average net years in dust hid the distribution of net years for single workers. Moreover, we also noted that there was no difference in lncRNA-ATB expression between healthy controls and healthy coal miners. It indicates that fibrosis, rather than dust exposure, might be related to lncRNA-ATB expression in plasma.

Recent evidence demonstrates that lncRNA-ATB also could serve as novel biomarkers for diagnosis and poor prognosis. Qu et al. reported that lncRNA-ATB was directly correlated with clinical endpoints (overall survival) and could serve as an independent prognosis maker for pancreatic cancer patients [15]. Saito et al. showed that increased lncRNA-ATB expression was a significant prognostic for increased recurrence and decreased survival of GC patients [26]. Iguchi et al. found that higher lncRNA-ATB was involved in the progression of CRC and was a novel indicator of poor prognosis in patients with CRC [14]. In this study, good AUCs of 0.84 when compared with healthy coal miners suggested that lncRNA-ATB may be used as a biomarker for detection of CWP. In addition, lncRNA-ATB was negatively associated with VC and FVC in CWP patients. These results indicated that upregulated lncRNA-ATB may participate in the progression of decreased lung function in CWP. In China, there are over 2.65 million coal mine workers, and over 12,500 new CWP patients were reported annually from 2010 to 2014 [32,33]. More preventive measures, such as being removed from dust exposed jobs, could be taken if pulmonary fibrosis could be detected in an earlier stage. Further investigation is needed to confirm the diagnosis role of lncRNA-ATB in big groups.

Our results had several major strengths. First, we evaluated the associations between lncRNA-ATB and clinical/biological features in CWP, and the results were comprehensively to explain the possible role of lncRNA-ATB. Moreover, all of the subjects were in the same place in China, minimizing the confounding effects of other characteristics, such as environment and socioeconomic factors. However, three limitations should also be addressed. First, in our study, we only observed the relationship between lncRNA-ATB and CWP, but the underlying mechanisms are unknown. Further studies are needed to explore potential mechanisms. Second, although the ROC curves were adjusted for potential confounders for plasma lncRNA-ATB, which permits a better differentiation between patients and controls, the unadjusted ROC curve may be more suitable in clinical practice. This needs further evaluation. Moreover, this study matched the average age among three groups, but the distribution of age was still a difference. Although we had adjusted age as a continuous variable in related analyses and found the trend of the results were similar after deleting subjects (age > 65). However, the role of age in the association between lncRNA-ATB and CWP is still needed to explore.

Overall, we discovered that lncRNA-ATB is significantly upregulated in CWP and positively associated with TGF-β1 in CWP patients. Moreover, elevated lncRNA-ATB was related with CWP risk and may be considered as a new biomarker for CWP in coal miners.

## 4. Materials and Methods

### 4.1. Study Population

We recruited 137 CWP patients and 72 healthy coal miners (net years in dust >15 years) from the Huangshi coal mine, which located in the central China, and 168 age-matched healthy controls recruited from the same city between November 2012 and June 2014. All subjects were male. The patients with CWP were diagnosed based on the China National Diagnostic Criteria for pneumoconiosis (GBZ 70-2009), which is consistent with the 1980 International Labor Organization on the classification of pneumoconiosis. In this study, we excluded the subjects with chronic diseases such as asthma, chronic obstructive pulmonary disease, pulmonary tuberculosis and cardiovascular disease. Trained investigators used a structured standardized questionnaire to collect information through face-to-face interviews, which included personal information, medical history, working history including net years in dust, and smoking status.

Approximately 5 mL of venous serum was collected from each participant and then put into a tube containing EDTA (Ethylenediaminetetraacetic acid). Plasma was obtained by centrifugation at 1500 rpm for 20 min and stored at −80 °C until use. Lung function tests were performed by a specialist using electronic spirometer (Chestgraph HI-101, CHEST Ltd., Tokyo, Japan). The lung function tests method were used as described in previous study [34]. Values used in this analysis included the percent of predicted FVC, percent of predicted FEV1, percent of predicted VC and % FEV1/FVC. This study was approved by the Ethics and Human Subject Committees of the Tongji Medical College Huazhong University of Science and Technology (Identification code: (2013) IEC (S017); date: 5 March 2013; Wuhan, China).

### 4.2. Total RNA Extraction and Quantitative Real-Time Polymerase Chain Reaction (qRT-PCR)

Total RNA from plasma was extracted by TRIzol LS Reagent (Life Technologies, Foster, CA, USA) according to the manufacturer’s protocol. After purification, RNA was extracted from 1.5 mL of plasma and dissolved in 25 μL of diethylpyrocarbonate (DEPC) water. The quantity and quality of the total RNA was determined with NanoDrop (Thermo, ND-1000, Waltham, MA, USA), and approximately 200 ng/µL RNA was obtained from the 1.5 mL plasma. Complementary DNA (cDNAs) was synthesized using Reverse Transcription Kit (TOYOBO, Osaka, Japan). qRT-PCR was performed to detect lncRNA-ATB expression by using the primer sequences as follows: F: 5′-CTTCACCAGCACCCAGAGA-3′ and R: 5′-AAGACAGAAAAACAGTTCCGAGTC-3′. GADPH was used as a gene reference. The primer sequences was as follows: F: 5′-CAGGAGGCATTGCTGATGAT-3′ and R: 5′-GAAGGCTGGGGCTCATTT-3′. cDNAs was amplified using the SYBR Green PCR Master Mix Kit (TOYOBO), and qRT-PCR was performed using the Applied Biosystems 7900HT Fast Real-Time PCR System (Life Technologies) according to the supplied manufacturer’s instructions. Relative quantification of RNA expression was calculated by using the 2^−∆∆*C*t^ method. Each sample was examined in triplicate.

### 4.3. Enzyme-Linked Immunosorbent Assay for Plasma Measurements (ELISA)

Plasma TGF-β1, MMP-2, MMP-9, Col-1 and Col-3 levels were measured by ELISA using commercially available kits. TGF-β1, MMP-9, MMP-2 assay ELISA kits purchased from R&D Systems Inc. (Minneapolis, MN, USA). Col-1 and Col-3 assay ELISA kits purchased from Uscn Life Science Inc. (Wuhan, China). All plasma samples were assayed in duplicate and the mean was calculated. The ELISA method processed manufacturer’s protocol.

### 4.4. Statistical Analysis

Comparisons were made by ANOVA test or Student’s *t*-test for variables with normal distribution. Least significant difference (LSD) was used to test for pairwise comparisons for normally distributed data. Kruskal–Wallis test was used for non-normally distributed data. Categorical variables were compared using a chi-Squared test. Spearmans’ correlation coefficients were calculated to determine the associations between lncRNA-ATB expression and clinical/biological features in patients. We further investigated the association between lncRNA-ATB expression and CWP risk. Compared with healthy controls, subjects were classified into three groups according to tertlile of lncRNA-ATB expression in the healthy controls. Then, compared with healthy coal miners, subjects were classified into three groups according to tertlile of lncRNA-ATB expression in the healthy coal miners. The classification of this study has been described previously [35]. Multivariable logistic regression models were performed to calculate ORs and 95% confidence interval (CI) for CWP according to the lncRNA-ATB expression. Mean ± standard deviation (SD) are reported for normally distributed data, unless stated otherwise. ROC curves were analyzed to assess the specificity and sensitivity of lncRNA-ATB for CWP. An optimal cut-off value was based on the Youden index. A two-sided *p*-value < 0.05 was considered statistically significant. Data were analyzed using the SAS, version 9.3, software (SAS Institute Inc., Cary, NC, USA).

## 5. Conclusions

In this study, we found lncRNA-ATB was significantly upregulated in CWP patients. Moreover, lncRNA-ATB may be considered as a new biomarker for CWP in coal miners.

## Figures and Tables

**Table 1:** Basic characteristics of the study population.

Variables	Normal Controls (*n* = 168)	Healthy Coal Miners (*n* = 72)	CWP Patients (*n* = 137)	*p*-Value
Age (years, mean ± SD)	58.83 ± 10.63	57.69 ± 10.86	59.27 ± 7.88	0.65 ^a^
Age (*n*, %)				
<55	45 (26.79)	54 (75.00)	40 (29.19)	<0.05 ^b^
≥55	123 (73.21)	18 (25.00)	97 (70.81)	
BMI (kg/m^2^, mean ± SD)	24.61 ± 3.33	25.50 ± 2.88	22.36 ± 3.46 ^#,^*	<0.05 ^a^
Net year in dust (*n*, %)				
≤30	/	62 (86.11)	90 (65.69)	<0.05 ^b^
>30	/	10 (13.89)	47 (34.31)	
Smoking status (*n*, %)				
Non-smoking	74 (44.05)	27 (37.50)	40 (29.20)	<0.05 ^b^
Smoking	94 (55.95)	45 (62.50)	97 (70.80)	
Blood pressure systolic (mm Hg, mean ± SD)	127.86 ± 21.51	n.d.	140.20 ± 23.76	<0.05 ^d^
Blood pressure diastolic (mm Hg, mean ± SD)	82.85 ± 16.67	n.d.	76.20 ± 13.06	<0.05 ^d^
FVC (% PRED FVC)	90.65 (64.90–120.30)	93.55 (71.40–109.40)	66.20 (28.20–104.50) ^#,^*	<0.05 ^c^
FEV1 (% PRED FEV1)	97.25 (67.10–95.44)	98.65 (89.00–111.20)	62.00 (17.70–108.40) ^#,^*	<0.05 ^c^
FEV1/FVC (% PRED)	80.75 (68.10–86.83)	83.94 (63.11–97.15)	74.81 (43.83–98.64) ^#,^*	<0.05 ^c^
TGF-β1 (pg/mL)	303.41 ± 28.38	425.64 ± 33.98 ^#^	569.99 ± 64.13 ^#,^*	<0.05 ^a^
Col-3 (ng/mL)	67.28 ± 7.79	70.26 ± 8.17	112.15 ± 9.16 ^#,^*	<0.05 ^a^
MMP2 (ng/mL)	142.27 (114.16–172.00)	166.73 (157.68–183.63) ^#^	206.32 (186.32–219.16) ^#,^*	<0.05 ^c^
MMP9 (ng/mL)	55.84 (45.03–68.81)	98.88 (75.03–112.32) ^#^	123.68 (105.16–143.27) ^#,^*	<0.05 ^c^
Col-1 (ng/mL)	28.65 (26.53–29.53)	29.90 (27.28–32.25)	34.15 (31.90.90–36.53) ^#,^*	<0.05 ^c^

Abbreviations: FVC, forced vital capacity; FEV1, forced expiratory volume in 1 s; % PRED FVC, percent of predicted FVC; % PRED FEV1, percent of predicted FEV1. BMI: body mass index. SD, standard deviation. ^a^ Calculated by ANOVA test, pair-wise comparisons calculated by Least significant difference; ^b^ Calculated by χ-Squared test; ^c^ Calculated by Kruskal–Wallis test; ^d^ Calculated by *t*-test; ^#^ Compared with Healthy controls, *p* < 0.05; * Compared with Healthy coal miners, *p* < 0.05; n.d., not done.

**Table 2:** Odds ratio of coal workers’ pneumoconiosis (CWP) according to the lncRNA-ATB expression.

Variables	LncRNA-ATB Expression Levels (Fold Change)			Per 1 Log-Unit Increment	*p*-Value *
	First Group <0.7559	Second Group 0.7559–1.1433	Third Group >1.1433		
**Healthy controls vs. CWP**					
**No. of cases/control subjects**	26/55	39/55	72/55		
**Model 1 (OR: 95% CI)**	1.00 (referent)	1.47 (0.79–2.74)	2.67 (1.49–4.78)	2.75 (1.69–4.48)	<0.05
**Model 2 (OR: 95% CI)**	1.00 (referent)	1.48 (0.80–2.75)	2.68 (1.50–4.80)	2.76 (1.70–4.49)	<0.05
**Model 3 (OR: 95% CI)**	1.00 (referent)	1.43 (0.75–2.74)	2.34 (1.28–4.30)	2.56 (1.54–4.27)	<0.05
**Model 4 (OR: 95% CI)**	1.00 (referent)	1.41 (0.73–2.72)	2.39 (1.29–4.42)	2.57 (1.52–4.33)	<0.05
**Healthy coal miners vs. CWP**	<0.7542	0.7542–1.2161	>1.2161		
**No. of cases/control subjects**	49/23	75/24	85/25		
**Model 1 (OR: 95% CI)**	1.00 (referent)	1.77 (0.85–3.70)	2.25 (1.08–4.68)	1.82 (1.05–3.17)	<0.05
**Model 2 (OR: 95% CI)**	1.00 (referent)	1.86 (0.84–4.15)	2.25 (1.02–4.97)	1.83 (1.01–3.35)	<0.05
**Model 3 (OR: 95% CI)**	1.00 (referent)	1.54 (0.64–3.70)	1.78 (0.74–4.31)	1.90 (0.97–3.72)	0.06
**Model 4 (OR: 95% CI)**	1.00 (referent)	1.47 (0.59–3.65)	2.05 (0.81–5.19)	2.25 (1.07–4.71)	<0.05
**Model 5 (OR: 95% CI)**	1.00 (referent)	1.65 (0.65–4.18)	1.90 (0.74–4.89)	2.17 (1.04–4.53)	<0.05

Model 1: single factor logistic regression. Model 2: adjusted for age (continuous). Model 3: adjusted for age (continuous), body mass index (BMI) (continuous). Model 4: adjusted for age (continuous), body mass index (BMI) (continuous), smoking status (no, yes). Model 5: adjusted for age (continuous), body mass index (BMI) (continuous), smoking status (no, yes), net years in dust (continuous). * *p*-values for the estimated changes by original log-transformed lncRNA-ATB expressions as a continuous variable.

**Table 3:** Correlations between lncRNA-ATB and clinical-/biological features in CWP patients ^†^.

Variables	LncRNA-ATB	TGF-β1	MMP-2	MMP-9	Col-1	Col-3	% PRED VC	% PRED FVC	% PRED FEV1	FEV1/FVC
LncRNA-ATB	1.00	0.30 *	0.07	−0.03	0.09	0.02	−0.18 *	−0.18 *	−0.10	0.05
TGF-β1		1.00	−0.04	0.09	0.18	−0.06	0.47 *	−0.55 *	−0.50 *	−0.30 *
MMP-2			1.00	0.04	0.09	−0.03	−0.02	−0.04	−0.08	−0.06
MMP-9				1.00	0.29	0.07	0.23 *	−0.20	−0.29 *	−0.31 *
Col-1					1.00	0.14	0.28 *	−0.25 *	−0.31 *	−0.20
Col-3						1.00	−0.15	−0.08	−0.13	−0.07
% PRED VC							1.00	0.93 *	0.88 *	0.47 *
% PRED FVC								1.00	0.90*	0.42 *
% PRED FEV1									1.00	0.75 *
FEV1/FVC										1.00

Abbreviations: VC, vital capacity; FVC, forced vital capacity; FEV1, forced expiratory volume in 1 s; % PRED VC, percent of predicted VC; % PRED FVC, percent of predicted FVC; % PRED FEV1, percent of predicted FEV1; ^†^ Adjusted for age (continuous), body mass index (BMI) (continuous), smoking status (no, yes), net years in dust (continuous); * *p* < 0.05.
